# Correlation of Nitrogen Sorption and Confocal Laser Scanning Microscopy for the Analysis of Amino Group Distributions on Mesoporous Silica

**DOI:** 10.3390/ma4061096

**Published:** 2011-06-09

**Authors:** Nando Gartmann, Dominik Brühwiler

**Affiliations:** Institute of Inorganic Chemistry, University of Zurich, Winterthurerstrasse 190, CH-8057 Zurich, Switzerland; E-Mail: nando.gartmann@aci.uzh.ch

**Keywords:** mesoporous silica, nanochannels, amines, silanes, distribution, confocal microscopy, nitrogen sorption

## Abstract

Aminopropylalkoxysilanes are frequently used for the functionalization of mesoporous silica. The analysis of amino group distributions on arrays of silica nanochannels by a combination of nitrogen sorption and confocal laser scanning microscopy provides valuable insight into the mechanisms underlying the interaction of these silanes with mesoporous silica surfaces. Tendencies towards external surface functionalization, non-uniform distribution in the pores, and hydrolysis of the silica framework are shown to depend to a large extent on the mobility of the aminopropylalkoxysilane molecules, which can be adjusted by the number and type of alkoxy groups.

## 1. Introduction

In the early 1990s, the synthesis of highly ordered mesoporous silica was first reported [1,2,3]. The following years saw further development towards providing a wide range of pore sizes and morphologies. Progress has been remarkable, leading to a rich palette of structure-directing agents (SDAs) and synthetic pathways [4,5]. The number of scientific publications on the topic of mesoporous silica literally exploded, mainly due to potential applications of these materials in fields as diverse as drug delivery [6,7] and catalysis [8]. Many of these applications require functionalization of the mesoporous silica, which is often conducted postsynthetically, for example by reaction with an alkoxysilane. In this context, interesting questions concerning the location of the functional groups on the mesoporous silica surface arise [9,10,11].

To investigate the parameters that affect the distribution of functional groups on mesoporous silica, we have been focusing on aminopropylalkoxysilanes, as they are among the most frequently employed reagents for the modification of mesoporous silica. Once the amines are anchored, a further moiety can be coupled by means of amine-reactive derivatives. This concept is especially useful for the visualization of functional group distributions by confocal laser scanning microscopy (CLSM), as it opens possibilities for fluorescent labeling [12,13,14]. Arrays of silica nanochannels (ASNCs) [15] fulfill the conditions required for the analysis of functional group distributions by CLSM and by nitrogen sorption, as the large, regularly shaped particles feature a comparatively narrow pore size distribution. The combined analysis by CLSM (single particle) and nitrogen sorption (ensemble) is particularly instructive concerning the interpretation of changes in pore size and pore volume upon surface functionalization. Not less importantly, it shows that an exclusive characterization by one of these two methods might lead to conclusions that are misleading.

## 2. Results and Discussion

**Figure 1:**
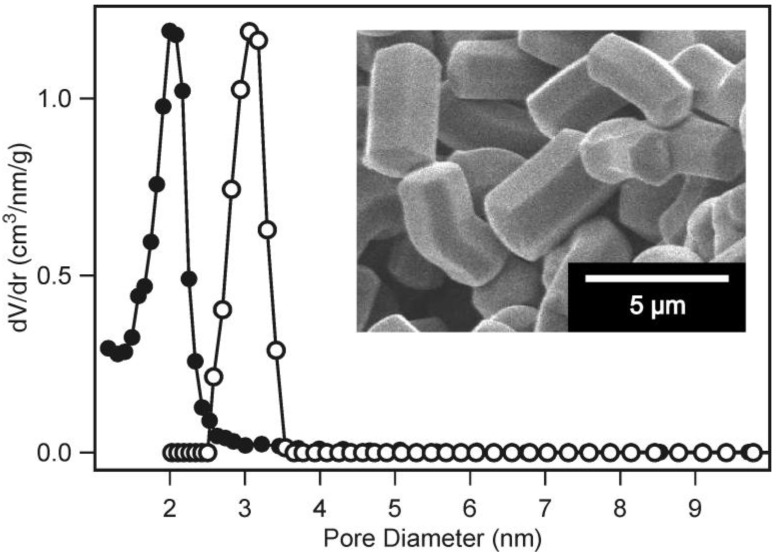
Pore size distributions of arrays of silica nanochannels (ASNCs) calculated from the nitrogen adsorption isotherm by BJH (●) and non-local density functional theory (NLDFT) (○). The inset shows a SEM image of calcined ASNCs.

ASNCs are hexagonally shaped fibers, each consisting of approximately 200,000 parallel nanochannels that run along the entire length of the particles [16]. As a consequence, the mesopore structure of ASNCs can be considered as a set of open-ended, non-intersecting cylinders. Calculation of the pore diameter from the adsorption isotherm by means of the BJH method [17] gives an average value of 2.02 nm. Whereas the standard BJH analysis is known to underestimate the pore size [18], a more reliable value of 3.06 nm is obtained by employing a non-local density functional theory (NLDFT) model (Figure 1) [19].

As the NLDFT kernel is not strictly valid for organo-functionalized silica surfaces, BJH was used to investigate the relative pore size changes upon grafting of the aminopropylalkoxysilanes. It should be noted that the structural properties of ASNCs (BET surface area, pore volume, pore diameter) show slight variation from batch to batch. For comparative studies such as the present one, it is therefore crucial to perform experiments with the same parent material.

**Figure 2:**
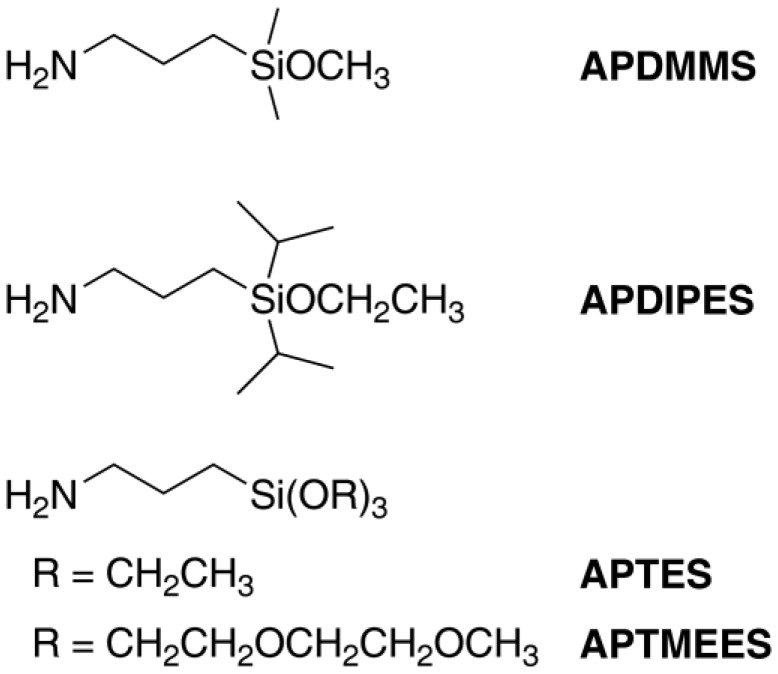
Structures and abbreviations of the employed aminopropylalkoxysilanes: 3-aminopropyldimethylmethoxysilane (APDMMS), 3-aminopropyldiisopropylethoxysilane (APDIPES), 3-aminopropyltriethoxysilane (APTES), and 3-aminopropyltris- (methoxyethoxyethoxy)silane (APTMEES).

**Figure 3:**
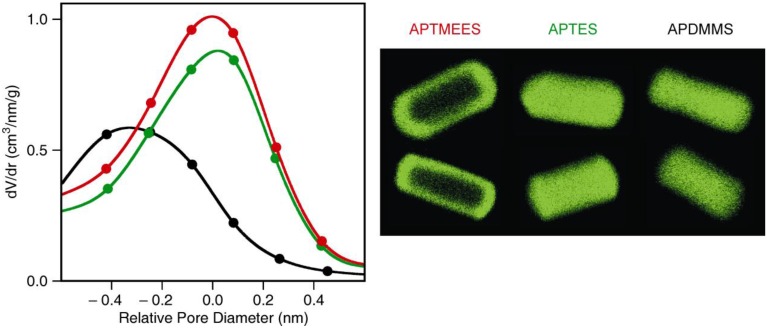
Representative confocal laser scanning microscopy (CLSM) images (after FITC labeling) of arrays of silica nanochannels (ASNCs) functionalized with different aminopropylalkoxysilanes (right panel) and the pore size distributions of the respective amino-functionalized materials relative to the pore diameter of the parent material: APTMEES (red), APTES (green), and APDMMS (black). The length of the particles in the CLSM images is in the order of 5 µm. Two particles are shown for each silane. Optical slices in the center of the particles were selected.

We have compared the grafting behavior of various aminopropylalkoxysilanes (Figure 2) by depositing an identical molar amount of each silane onto ASNCs from toluene at room temperature. The samples were labeled with fluorescein isothiocyanate (FITC) and imaged by CLSM. Structural properties of the materials are given in Table 1. CLSM images of the FITC-labeled materials along with the pore size distributions of the amino-functionalized samples are shown in Figure 3. In all experiments, the grafting behavior of APDIPES was found to be very similar to that of APDMMS. For brevity, only the results for APDMMS are shown.

**Table 1:** BET surface area (S_BET_) and pore volume (V_tot_) of parent and amino-functionalized arrays of silica nanochannels (ASNCs).

	S_BET_ [m^2^/g]	V_tot_ [cm^3^/g]
parent	1170	0.72
APDMMS	865	0.44
APTES	987	0.61
APTMEES	1115	0.69

Deposition of APTMEES causes only a minor decrease of the pore volume, pore diameter, and BET surface area (Table 1, Figure 3). This is commonly interpreted as being a consequence of external surface functionalization. Indeed, CLSM images of the fluorescent-labeled samples confirm this interpretation and are in agreement with previously reported results [12].

In the case of APTES, pore volume and BET surface area are significantly reduced upon deposition, suggesting derivatization of the pore surface. The corresponding CLSM images support this conclusion by showing fluorescence over the entire length of the particles. It is interesting to note that the maximum of the pore size distribution of the APTES-functionalized sample remains at a pore size value similar to that of the parent material. This typically indicates a non-uniform distribution of the grafted moieties in the pores, with the pore body having a lower functionalization degree than the pore surface close to the pore entrances. For APTES, however, this effect does not seem to be pronounced enough to become visible in the CLSM images.

To our surprise, deposition of APDMMS strongly reduced the BET surface area, pore volume, and pore diameter. This could be interpreted as a homogeneous distribution of the grafted amino groups over the entire pore surface. While such an assumption is supported by the corresponding CLSM images, as well as by the fact that monoalkoxysilanes are known to produce more uniform distributions than trialkoxysilanes [20], the effect is rather large for the comparatively low grafting densities investigated in this work. Fluorescamine analysis yielded an amino content of 50 µmol/g after deposition of APDMMS. This corresponds roughly to 1200 amino groups per nanochannel or 0.025 amino groups per nm^2^.

**Figure 4:**
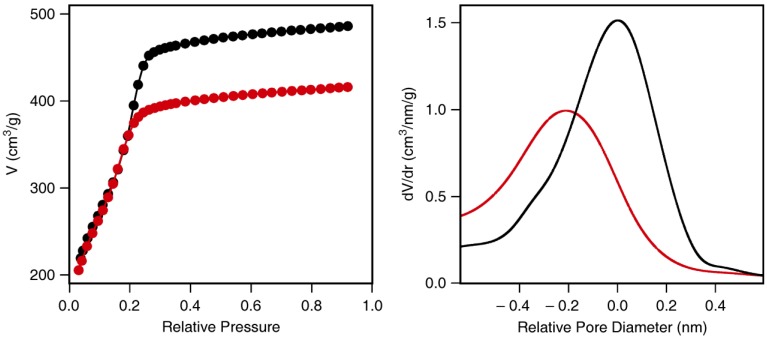
Nitrogen adsorption isotherms (left) and corresponding pore size distributions (right) of arrays of silica nanochannels (ASNCs) before (black) and after deposition and removal of n-hexylamine (red).

To gain insight into the mechanisms leading to the pronounced pore size reduction observed for APDMMS, we conducted blind experiments by replacing APDMMS with n-hexylamine. This allowed us to focus on possible effects of the amino group in the absence of covalent bond formation. As expected, the final product only contained negligible amounts of amino groups (less than 2 µmol/g) after washing with ethanol and 0.4 M aqueous HCl to remove the electrostatically adsorbed n-hexylamine molecules. Surprisingly, despite being a non-functionalized product, the pore size distribution after removal of n-hexylamine is shifted towards smaller pore size (Figure 4), indicating a partial hydrolysis of the silica framework. The fact that this effect was only observed for n-hexylamine, APDIPES, and APDMMS, points to the important role of the mobility of the respective amine. Our samples most likely contained a certain amount of trace water. When depositing silanes from solution, trace water is generally difficult to eliminate, as silica behaves as an efficient drying agent, adsorbing even minute quantities of water [21]. Under our conditions used for grafting, we can expect these adsorbed water molecules to be immobile and localized [22,23]. Hydrolysis of the silica framework is therefore only promoted if the amine is sufficiently mobile. APTMEES molecules react with silanol groups on the external surface and are quickly immobilized, leaving the mesoporous silica framework intact. From this set of experiments we can conclude that the high mobility of APDMMS and APDIPES leads to uniform distributions of grafted amino groups, but also causes partial hydrolysis of the silica framework.

## 3. Experimental Section

### 3.1. Arrays of Silica Nanochannels

ASNCs were synthesized according to a published procedure [15]. However, as our comparative studies require larger batches of starting material, the procedure was upscaled by a factor of 3. Briefly, an amount of 14.55 g of hexadecyltrimethylammonium chloride (Acros, 99%) was dissolved in 228 mL of double distilled H_2_O and 180 mL of 32% aqueous HCl by stirring for 1 min at *ca*. 1000 rpm in a polypropylene beaker. The solution was subsequently cooled to 0 °C for 15 min without stirring, followed by the slow addition of 6 mL of cold tetraethoxysilane (Aldrich, 99.999%) and further stirring for 30 s. The resulting mixture was kept at 0 °C under quiescent conditions for 3 h. The product was collected by filtration and washed with H_2_O. The SDA was removed by first heating at 300 °C for 2 h and calcining at 550 °C for 12 h. Heating rates of 2 °C/min were applied.

### 3.2. Functionalization

Reactions of aminopropylalkoxysilanes with ASNCs were conducted by dispersing 200 mg of ASNCs in 10 mL of dry toluene and subsequently adding 20 µmol of the respective silane. After the mixture had been stirred for 10 min at room temperature, the functionalized ASNCs were recovered by filtration and cured in an oven at 80 °C for 16 h.

### 3.3. Characterization

Labeling with fluorescein 5-isothiocyanate (FITC, Fluka, ≥ 97.5%) was carried out according to [20], with a coupling time of 24 h at room temperature (in absolute ethanol). The amount of surface-grafted amino groups was analyzed by the fluorogenic derivatization reaction with fluorescamine [24]. Nitrogen sorption isotherms were collected at 77 K using a Quantachrome NOVA 2200. Samples were vacuum-degassed at 80 °C for 3 h. The total surface area S_BET_ was obtained using the standard BET method for adsorption data in a relative pressure range from 0.05 to 0.10 [25]. The total pore volume V_tot_ was calculated from the amount of nitrogen adsorbed at a relative pressure of 0.95. The relative changes of the mesopore size distributions upon functionalization were evaluated by analyzing the adsorption isotherms by means of the BJH model [17]. A NLDFT model developed for silica exhibiting cylindrical pore geometry (NOVAWin2 software, Version 2.2, Quantachrome Instruments) was employed to characterize the parent materials [19]. Scanning electron microscopy images were acquired on a JEOL JSM-6060. The CLSM setup consisted of an Olympus BX 60 microscope with a FluoView confocal unit. The FITC-labeled samples were excited at 488 nm. Optical slices in the center of the particles were selected.

## 4. Conclusions

The characterization of porous materials by nitrogen sorption is a standard technique to obtain information on BET surface area, pore volume, and pore diameter. When investigating the outcome of grafting reactions, the interpretation of the respective data in terms of the location of the grafted moieties is often difficult and results tend to be ambiguous. We have shown that complementing nitrogen sorption with CLSM imaging greatly facilitates the interpretation of pore structure data. The combination of nitrogen sorption and CLSM paints a comprehensive picture of the distribution of the amino groups on ASNCs after functionalization with various aminopropylalkoxysilanes. Tendencies towards external surface functionalization, non-uniform distribution in the pores, and hydrolysis of the silica framework can be identified and were shown to depend to a large extent on the mobility of the respective aminopropylalkoxysilane molecules.
